# SPR-Based Sensor for the Early Detection or Monitoring of Kidney Problems

**DOI:** 10.1155/2022/9135172

**Published:** 2022-06-16

**Authors:** Budi Mulyanti, Harbi Setyo Nugroho, Chandra Wulandari, Yuni Rahmawati, Lilik Hasanah, Ida Hamidah, Roer Eka Pawinanto, Burhanuddin Yeop Majlis

**Affiliations:** ^1^Department of Electrical Engineering Education, Universitas Pendidikan Indonesia, Bandung 40154, Jawa Barat, Indonesia; ^2^Department of Physics Education, Universitas Pendidikan Indonesia, Bandung 40154, Jawa Barat, Indonesia; ^3^Engineering Physics, Institute Teknologi Bandung, Bandung 40132, Jawa Barat, Indonesia; ^4^Department of Mechanical Engineering Education, Universitas Pendidikan Indonesia, Bandung 40154, Jawa Barat, Indonesia; ^5^Institute of Microengineering and Nanoelectronics (IMEN), Universiti Kebangsaan Malaysia (UKM), Bangi 43600, Selangor, Malaysia

## Abstract

SPR-based technology has emerged as one of the most versatile optical tools for analyzing the binding mechanism of molecular interaction due to its inherent advantages in sensing applications, such as real-time, label-free, and high sensitivity characteristics. SPR is widely used in various fields, including healthcare, environmental management, and food-borne illness analysis. Meanwhile, kidney disease has grown to be one of the world's most serious public health problems in recent decades, resulting in physical degeneration and even death. As a result, several studies have published their findings regarding developing of reliable sensor technology based on the SPR phenomenon. However, an integrated and comprehensive discussion regarding the application of SPR-based sensors for detecting of kidney disease has not yet been found. Therefore, this review will discuss the recent advancements in the development of SPR-based sensors for monitoring kidney-related diseases. Numerous SPR configurations will be discussed, including Kretschmann, Otto, optical fiber-based SPR, and LSPR, which are all used to detect analytes associated with kidney disease, including urea, creatinine, glucose, uric acid, and dopamine. This review aims to show the broad application of SPR sensors which encouraged the development of SPR sensors for kidney problems monitoring.

## 1. Introduction

Chronic kidney disease (CKD) has been one of the most severe public health issues in recent decades, with a global prevalence of about 11–13% (majority at CKD stage III), and patients with CKD stage V or end-stage renal disease (ESRD) require a complicated, limited, and expensive treatment such as hemodialysis or kidney transplant [1, 2]. According to global estimates, approximately 4.902 to 7.083 million people require kidney transplant surgery [3]. From 2010 to 2017, the number of ESRD and prevalent cases in Indonesia increased significantly, from 9649 to 30831 cases and from 11484 to 77892 cases, respectively [4]. The kidneys are vital organs that never stop filtering the blood of numerous wastes and toxic substances and removing them via urine while returning essential substances such as vitamins, amino acids, glucose, and hormones to the bloodstream [5]. Because the kidneys have been damaged either physically or through disease, it would be detrimental to the body's toxins and excess fluids. Diabetes and hypertension are the most common diseases that cause kidney damage [6–8]. Lethargy, swelling, persistent headaches, lower back pain, fluid retention, seizures, coma, and eventually death are all symptoms of damaged kidneys [9, 10]. As a result, an early detection strategy and proper CKD management are required to reduce the prevalence and incidence of ESRD, thereby lowering morbidity and mortality rates as well as the healthcare system's cost.

Problems with the kidneys are frequently associated with an increase in metabolic waste in the bloodstream. Thus, urea and creatinine become the primary indicators for kidney problems [11]. In addition, elevated levels of glucose, uric acid, and dopamine can also indicate kidney disease. Diabetic kidney disease is a kidney disease caused by diabetes which is known as a disease due to high levels of glucose in the blood [12]. Meanwhile, uric acid is one of the products excreted by the kidneys as the end product of purine metabolism oxidation [13]. Dopamine also serves essential functions in the kidney by regulating net salt and water excretion and activating intrarenal dopaminergic pathways to prevent hypertension [14]. Numerous researchers have developed various methods for the early detection of kidney disease and blood monitoring systems during hemodialysis procedures in recent years. It consists of electronic skin (E-skin) [15], optical ultracompact [16], ultrasensitive sensor [17], supercapacitive hybrid sensor [18], molecularly imprinted polymers (MIP) [19], solid-state sensor [20], conductometry [21], potentiometry [22], spectrometry [23], matrix-assisted laser desorption/ionization mass spectrometry (MALDI-TOF MS) [24], and high-performance liquid chromatography (HPLC) [25]. Among these numerous methods, SPR stands out as one of the most promising and attractive candidates for development due to its primary advantages of label-free, highly efficient, and real-time measurement [26–30]. As a result, SPR is an extremely advanced and powerful technique for studying the kinetic properties of molecular interactions by directly measuring molecular interactions and chemical bonding in real-time without the use of labels [31–33].

The research on optical sensors based on surface plasmon resonance (SPR) has grown significantly over the years, evolving from a relatively unknown physical phenomenon to a well-known optical tool for observing the binding mechanism of molecular interaction, with over 24,000 publications by 2019 [34]. SPR occurs when incident light with a specific incidence angle excites electrons on a metal surface and then propagates through the interface between the metal layer and the dielectric material [35]. The resonance peak appears as the reflectance decreases due to energy transfer via a plasmonic wave from the incident light to the dielectric medium adjacent to the metal surface [36, 37]. The presence of a particular molecule (analyte) on a metal surface results in changes in the metal surface refractive index (RI), which results in an angular shift of the resonance peak [34, 38]. SPR's primary advantage is its ability to detect molecular phenomena in real-time, which enables rapid diagnostic sensing. Real-time sensing is accomplished by alternately switching between adsorption and desorption kinetics and measuring the dip angle changes each time [26, 34, 39].

In general, SPR has been used to detect a variety of targets, including glucose [40–42], uric acid [43, 44], dopamine [27, 45, 46], antibody [47–50], antigen [47, 51, 52], salmonella [53], urea [43, 54–56], and creatinine [28, 57, 58], in a variety of applications, including material characterization, medical diagnostics, food quality sensors, and drug development. Yu et al. described the development of a D-shaped fiber SPR glucose biosensor with a modified MoS2-graphene composite nanostructure that exhibits approximately fourfold increased sensitivity [40]. The excellent photoelectric properties were attributed to a composite nanostructure formed via liquid phase transfer, resulting in high glucose detection selectivity. Hallaj et al. developed a dual-mode sensor for detecting uric acid in human urine samples using N, P-CDs, and AuNPs/Ag+ [59]. The hydrothermal method was used to synthesize green emissive N, P-CDs. Meanwhile, sensor design was based on in-situ Au@AgNPs formation and the inner filter effect (IFE). Additionally, double sensitivity to dopamine 10^−12^ M was achieved using SPR sensors based on a thin film of perylene bisimide (PBI)/Au nanostructures (AuNs) [45]. The PBI/AuNS hybrid layer can enhance the interfacial coupling of plasmonic waves propagating on AuNs. Menon et al. used an SPR-based sensor to investigate the detection of urea and creatinine in nonenzymatic samples with varying concentrations [55]. Urea and creatinine were successfully detected at concentrations ranging from 50 to 800 mM and 10 to 200 mM, respectively. These numerous SPR sensor applications demonstrate the technology's utility, which has fueled the development of SPR sensors for various detection targets.

A comprehensive review of SPR-based sensors for kidney disease markers is required to contribute to the study of kidney disease early detection or monitoring. Numerous studies will be discussed to provide readers with a comprehensive and in-depth understanding of the development, modification, and recent progress of SPR-based sensors for kidney disease. The discussion will begin with the relationship between the markers level and kidney disease before moving on to the theory and operation of SPR-based sensors. We will discuss the plasmonic and metal layers used in SPR sensors. SPR sensors have a plasmonic and metal layer comprised of thin-film material and an immobilization layer. The discussion will then focus on the performance of SPR sensors for detecting urea, creatinine, glucose, uric acid, and dopamine. Finally, a summary and future directions for SPR sensors for kidney disease markers detection will be presented in the pursuit of reliable, effective, and efficient tools for the early detection and monitoring of kidney problems.

## 2. The Markers of Kidney Diseases

The kidneys are a vital organ that removes waste and excess water from the blood via urine, maintains crucial chemical and electrolyte balance through reabsorption, regulates the acid-base balance, and functions independently of the nephron to activate vitamins and synthesize hormones [60]. Healthy the human body suffers once the kidneys work nonstop to carry out their life-sustaining function of filtering blood in the human body. However, the human body suffers once the kidneys are damaged, whether through disease or physical trauma. Damaged kidneys result in CKD, which, if left untreated, can progress to ESRD or kidney failure [6–8]. This issue will eventually result in a variety of negative consequences, including nausea, weakness, swelling, shortness of breath, and poor sleep, as a result of the body's waste products and fluid accumulation [9, 10]. Finally, if the condition is not treated seriously and the kidneys cease to function thoroughly, it can be fatal [9, 10]. Once the kidneys fail, it is necessary to undergo dialysis or a kidney transplant [1, 2].

Furthermore, the global ranking of CKD as a cause of death has risen significantly in the last two decades, with approximately 10% of the global population affected by CKD and unable to obtain affordable treatment [9, 61]. It has been reported that more than 2 million people worldwide receive dialysis or kidney transplants, but only about 10% of the total population requires it [62]. As a result, early detection technology is critical for kidney problems, particularly in the early stages of CKD, to prevent progression into more dangerous and complicated issues. The use of biochemical markers in serum analysis is critical for detecting kidney disease [63, 64]. Urea and creatinine are the most commonly used biochemical markers because they represent the glomerular filtration rate (GFR), which defines kidney function [65]. Urea and creatinine concentrations in human blood are highly related to kidney function because they are waste products from protein metabolism carried to the kidneys alongside blood to be filtered and removed. As a result, if their concentration increased, it could indicate a kidney problem [66]. Urea and creatinine concentration ranges in human blood in a healthy body are 2.5–6.7 mM and 35–140 M, respectively [67, 68]. Meanwhile, an unhealthy body raises urea and creatinine levels to 30–150 mM and 1000 mM, respectively [67, 68].

Apart from urea and creatinine, several analytes such as glucose, uric acid, and dopamine can also be used to diagnose kidney disease [12]. Additionally, the glucose level indicates the condition that may result in kidney problems, or is named diabetic kidney disease. A high concentration of glucose in the blood, typically >126 mg/dL during fasting blood test, indicates types 2 diabetes. A person with diabetes condition is prone to kidney problems since a high glucose concentration in the blood could damage the blood vessels in the kidneys [69]. In recent years, uric acid has been proposed as a crucial factor in the pathophysiology of chronic kidney disease and possibly acute kidney damage [13]. The presence of uric acid concentration above the normal level of 200 mg/L at pH of 5.3 can lead to the formation of kidney stones which can damage the kidneys and block the waste removal process [70]. The dopamine was assisting the kidney to prevent hypertension by controlling net salt and water excretion as well as activating intrarenal dopaminergic pathways. The lower dopamine concentration in the human body could lead to hypertension, narrowing blood vessels in the entire body, including the kidneys [14]. As a result, kidneys no longer work properly.

## 3. Surface Plasmon Resonance (SPR): Theory and Working Principle

**Figure 1:**
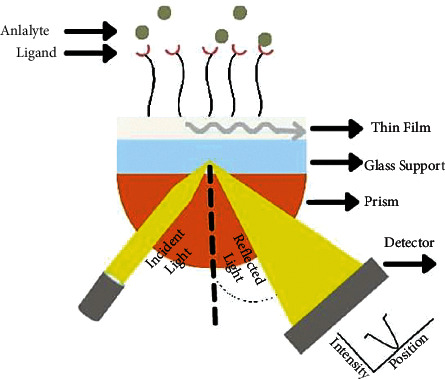
Illustration of surface plasmons resonance (SPR).

Figure 1 depicts the structure and operation of SPR sensors. The Kretschmann configuration is the most commonly used SPR configuration [71]. A p-polarized light with a specific incident angle is typically reflected into the photodetector after passing through a prism to the sensing surface. The sensing surface is commonly made up of a thin metal or bimetal film that generates the plasmonic waves [72–75]. Surface plasmons, or a collective oscillation of conduction electrons from a thin film, resonate with incident light at a specific angle known as the resonance angle, causing energetic plasmonic waves to be generated at the thin-film/dielectric interface [36]. However, because the incident light's propagation constant is always lower than that of the plasmonic wave, the incident light cannot directly excite the plasmonic waves at a planar metal-dielectric interface [76]. Therefore, the prism is used to increase the momentum of incident light via attenuated total reflection (ATR) within the prism and diffraction at the diffraction gratings surface. Plasmonic waves generate an exponentially decaying electric field known as an evanescent field, which interacts with the surrounding dielectric medium at a penetration depth of hundreds of nanometers [35]. As a result, the evanescent field is susceptible to changes in the refractive index of the sensing surface caused by the binding of ligand and molecule (analyte), resulting in a resonance angle shift of 2.5. The resonance angle shift is then used to characterize the performance of SPR-based sensors in the presence of an analyte.

A ligand (e.g., enzyme, antibody) is coupled to a thin film in order to capture the analyte target (enzyme-substrate, antigen, or complementary DNA) presented on the sensing surface [77]. As such, during the fabrication process, an immobilization matrix is frequently coated on the sensing surface of thin films [26]. The immobilization matrix immobilize the ligand without impairing its functionality, also can be used to improve sensor performance [78–81]. Numerous materials have been reported for use as an immobilization matrix, including MoS2, graphene, silicon, and MOFs [40, 82–84]. Wang et al. demonstrated that the presence of a two-dimensional MoS2 nanosheet increased the sensitivity of the SPR sensor by increasing the evanescent field and aiding in the immobilization of the ligand on the sensing surface [85]. Additionally, Hossain et al. reported that inserting a graphene layer can aid in improving the evanescent field distribution, as graphene possesses excellent plasmonic and optical absorption properties [86].

The optimal SPR sensing performance is characterized by a deep and sharp reflectance that is proportional to a low reflectivity and a full width at a half maximum (FWHM) value of 6. Optimizing the thickness of thin films reduces reflectivity by increasing energy transfer from incident light to surface plasmons [87]. A small FWHM value indicates the sharpness of an SPR reflectance curve obtained from a dielectric constant with a small imaginary part and a significant real part. Sensitivity, the figure of merit (FOM), and limit of detection (LOD) are three parameters used to evaluate the performance of an SPR-based sensor. Sensitivity is determined by the shift in the resonance angle caused by the refractive index changes caused by the presence of the analyte near the sensing surface. The FOM is calculated by dividing the sensitivity value by the FWHM. Finally, the LOD establishes the lowest detectable concentration of the targeted analyte. The LOD can be calculated as LOD = ^∆^*λ*/S, where ^∆^*λ* is the wavelength resolution of the spectrometer and S is the sensor sensitivity [85].

### 3.1. SPR-Based Sensor for Detection of Kidney Problems

The rise in prevalence of kidney diseases such as CKD and ESRD among global public health concerns have resulted in significant problem, which has resulted in several negative consequences, including weakness, swelling, poor sleep, nausea, coma, and eventually death [1–4]. Patients with these complications will require dialysis or a kidney transplant [1, 2]. Thus, in recent years, interest in developing medical technology for early detection or monitoring kidney problems has grown. Identifying the detection variable (analyte) for kidney problems is necessary to initiate or monitor treatment. Among various markers, urea and creatinine are the most frequently used analytes for diagnosing and monitoring kidney problems due to their ability to define kidney function by representing the glomerular filtration rate (GFR) [65]. Increased urea and creatinine levels in human blood indicate kidney problems [66].

**Figure 2:**
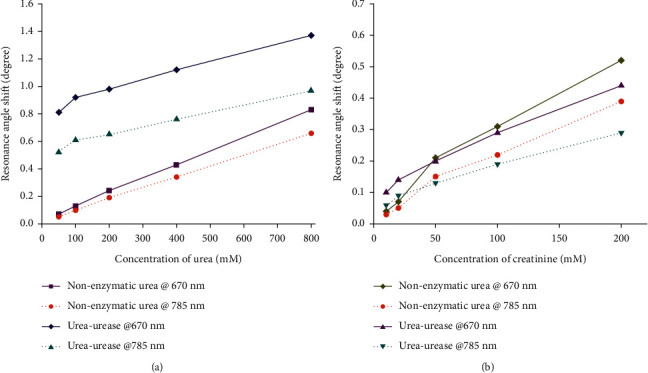
The presence of ligand increases resonance angle shift.

Table 1 summarizes reports of SPR-based sensors for a variety of detection targets associated with kidney problems. Menon et al. used Kretschmann-based 50 nm thick gold film SPR sensors to compare nonenzymatic and enzymatic samples for urea and creatinine detection [55]. Urease and creatinase are used in the enzymatic samples to detect urea and creatinine, respectively. This enzyme was used as a bioreceptor, detecting specific analytes and thus improving sensor selectivity. The enzymatic samples were prepared by mixing the urea and creatinine with urease and creatinase in an aqueous solution, respectively. Urea and creatinine detection concentration ranges are 50–800 mM and 10–200 mM, respectively. Nonenzymatic samples have sensitivities of 1.4 × 10^−3^°/M and 4 × 10^−3^°/mM to 670 nm wavelength exposure for urea and creatinine, respectively. The enzymatic samples have sensitivities of 1.62 × 10^−2^°/mM for urea-urease samples and 1 × 10^−2^°/mM for creatinine-creatinase samples. The increased sensitivity for both enzymatic samples is attributed to urease and creatinase acting as a ligand for urea and creatinine analytes, which aids in capturing analytes to the sensing surface. As illustrated in Figure 2, the presence of ligand results in a greater shift of the resonance angle, resulting in increased sensitivity for the device [55].

Pothipor et al. developed a novel nonenzymatic SPR sensor for creatinine detection using starch-stabilized silver nanoparticles (starch-AgNPs) on poly(pyrrole) (PPy) Au thin film [57]. At a creatinine detection range of 0.01–1 mM, the best sensitivity was determined to be 86.3 cm^−2^ *μ*M with a LOD of 1.9 × 10^−4^ mM. The presence of AgNPs contributes to the sensing surface's increased sensitivity and selectivity for creatinine samples. Additionally, AgNPs contribute to the enhancement of absorption via localized plasmon effects. Meanwhile, Topçu et al. modified the gold surface of SPR sensors with functional monomers derived from N-methacryloyl-(l)-histidine methyl ester (MAH) to detect creatinine [28]. The modification was accomplished through the molecular imprinting technique, which resulted in the creation of a synthetic creatinine receptor on the gold surface. The SPR sensor used in this work has a detection range of 1 to 100 mM and a limit of detection of 57 and 190 mM, respectively. Another type of SPR is local surface plasmon resonance (LSPR) [88, 95]. Recent advances in nano-optics have paved the way for developing highly sensitive and mark-free optical transducers based on the surface plasmon resonance (SPR) of metallic nanostructures. LSPR is an optical phenomenon caused by electrons collectively oscillating in metal nanostructures surrounded by a dielectric [88, 95]. The metal nanostructure absorb a portion of the incident light, while the remainder is scattered in various directions. Chamuah et al. enhanced the ability of the surface-enhanced Raman scattering (SERS) with Blu-Ray DVD (BRDVD) substrate to detect urea and creatinine in urine samples by inserting AuNPs to perform the LSPR phenomenon [88]. The lowest urea and creatinine concentrations detected in this work are 0.6 and 0.2 g/mL, respectively. These values are within the range of abnormal urea and creatinine concentrations in urine samples, indicating that the device can be used for clinical purposes. By generating both the photon lifetime of the coupled electromagnetic field and the guided-mode resonance (GMR) field, the presence of AuNPs in the BRDVD channel increases the local field intensity. Additionally, this work achieved a high SERS signal stability over a 45-day period.

**Figure 3:**
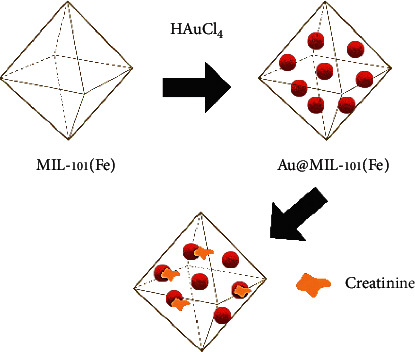
The schematic in-situ construction and the creatinine capturing mechanism of Au@MIL-101(Fe).

In another study, metal-organic frameworks (MOFs) are used as LSPR sensors, with AuNPs serving as the metal clusters and joints and MIL-101(Fe) serving as the organic linkers [89]. Coordination bonds and molecular interaction between metal joints and organic linker struts form the interconnected network. MOFs have high catalytic activity, large surface area, anda variety of bonding interactions with analytes, making them an attractive candidate for sensing applications. Jiang et al. fabricated Au@MIL-101(Fe)-based SERS sensors with the in-situ growth of AuNPs in MIL-101(Fe) [89]. The device is extremely sensitive to low creatinine concentrations in urine, with a LOD of 1 × 10^−4^ mM and a detection range of 10^−3^—10^−1^ mM. Following the manifestation of Au@MIL-101(Fe), the octahedral structure is partially broken down by the embedding of AuNPs into MIL-101(Fe), as illustrated in Figure 3. Taking advantage of electrostatic force to enrich the porous MIL-101(Fe) structure, the significantly improved Raman scattering signal was attributed to creatinine molecules approaching closer to the AuNPs vicinity and thus increasing the Raman scattering signal (d). The accumulation of metals within MOFs is thought to be responsible for the abundance of LSPR hot spots, as MIL-101(Fe) could serve as a suitable platform for the binding of AuNPs [89].

Numerous studies have reported on the combination of SPR and LSPR configurations. The structure consists of an optical fiber coated with a thin metal layer, which is then covered with metal nanoparticles. This configuration enhances the sensing ability of both SPR and LSPR mechanisms [54, 56]. Verma et al. developed a novel design for urea detection using the gel entrapment technique in an optical fiber multianalyte sensor [56]. By coating the fiber optic in channel 1 with an Ag/Si layer, sensing probes are prepared. They discussed the numerous advantages of their work, including miniaturized probes, simplicity, low cost, quick response, ease of handling, and online monitoring.

**Figure 4:**
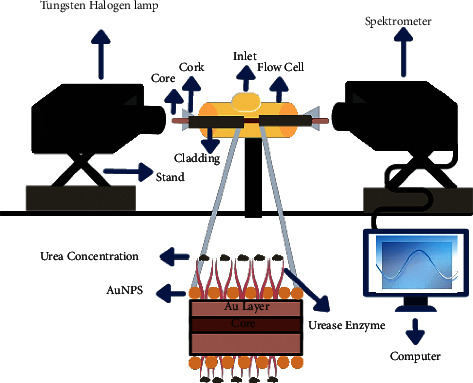
The schematic experimental setup configuration of the multimode optical fiber-based sensor utilizing SPR and LSPR phenomenon for urea detection.

Similarly, Gupta et al. employed the SPR/LSPR phenomenon to develop a multimode optical fiber-based sensor for the detection of enzymatic and nonenzymatic urea [54]. To modify the sensing probe, biosynthesized AuNPs were coated onto the optical fiber coat Au thin film.  Figure 4 depicts the device configuration used in this work. The presence of AuNPs increased sensitivity by twofold and threefold, respectively, for nonenzymatic and enzymatic urea samples, when compared to the probe without AuNPs. Sensitivity values for nonenzymatic and enzymatic urea samples are 0.04537 and 0.06013 nm/mM, respectively, over the detection range of 50 to 800 mM [54].

**Figure 5:**
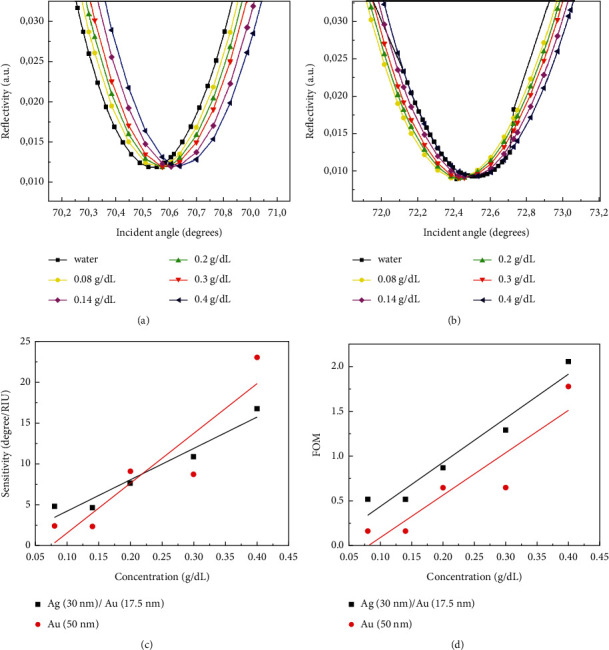
SPR response curve with a different Kretschmann configuration of a (a) 30 nm·Ag/17.5 nm·Au thin film and (b) 50 nm Au thin film for different glucose concentration, and a corresponding performance comparison in (c) sensitivity and (d) figure of merit (FOM).

Determining glucose concentrations in human blood is critical for medical and human health monitoring. A high glucose concentration in the human body causes several dangerous conditions, including hypoglycemia and diabetes [96, 97]. Diabetes is caused by a higher glucose concentration in the human body, whereas hypoglycemia is caused by a lower concentration in the human blood. As a result, a high-sensitivity sensor for measuring glucose concentration in the human body is required. Yu et al. described a D-shaped fiber SPR glucose biosensor with a modified MoS_2_-graphene composite nanostructure that has a fourfold increase in sensitivity [40]. The excellent photoelectric properties were attributed to a liquid phase transfer-induced composite nanostructure, resulting in a high selectivity for glucose detection. Zheng et al. improved the performance of optical-based glucose sensors by combining the SPR phenomenon with a glucose oxidase (GOD) sensitive film [42]. To generate the SPR phenomenon, a gold thin film was coated onto the surface of an optical fiber, and then a glucose oxidase (GOD) sensitive film was covalently bonded to the gold thin film. The resonance angle shift was nearly linear between 0 and 2.8 mM but began to turn exponentially once the concentration exceeded 2.8 mM. The nearly linear resonance angle shift could occur because the quick binding of the glucose molecule to the enzyme due to enzyme binding sites is sufficient for a low glucose concentration. However, as the glucose concentration rises, the enzyme content decreases, slowing the binding velocity between enzyme and glucose molecule, resulting in a slower resonance angle shift and nonlinear fit. The device used in this study had a high sensitivity of 15.38 nm/mM within a detection range of 0–2.8 mM and a detection limit of 2.2 × 10^−3^ mM. Furthermore, the device has good selectivity and stability, which is promising for biomedicine and human health monitoring. The modification of metallic thin film also can be obtained by use of bimetallic film as reported by Mulyanti et al. [98]. In Figures 5(a) and 5(b), the SPR response curve of 30 nm·Ag/17.5 nm·Au thin film and 50 nm·Au -based SPR, respectively, in the detection of different glucose concentration resulted in the shifting which indicates the sensitivity. The comparison concluded better sensitivity and figure of merit (FOM) obtained by the bimetallic SPR sensors, as shown in Figures 5(c) and 5(d), respectively [98].

Additionally, the concentration of uric acid in the human body must be examined and monitored. A high uric acid level in the bloodstream may indicate a variety of health problems, including diabetes, leukemia, gout, limb swelling, lead poisoning, and metabolic acidosis [99]. As a result, numerous researchers have been working on developing new sensor technology to address these issues. Kant et al. developed a fiber optic sensor based on LSPR for the detection of uric acid in aqueous samples [91]. To fabricate the sensing probe, successive layers of silver and silicon were coated over a small unclad length of silica optical fiber. The uricase enzyme was then immobilized using the gel entrapment method by dipping the sensing probe into a polyacrylamide gel containing the uricase enzyme. The sensor's performance was evaluated over the detection range of 0–0.9 mM uric acid, resulting in a sensitivity and limit of detection (LOD) of 10.5 nm/mM and 0.032 mM, respectively. Jain et al. recently developed an optical sensor for uric acid detection based on Long-Range Surface Plasmon Resonance (LR-SPR) with an Otto configuration [92]. Due to its low and tunable refractive index, silicon dioxide (SiO_2_) is used as a dielectric layer for LR-SPR mode excitation instead of the more commonly used Cytop and Teflon polymers. The LR-SPR sensor structure was evaluated in the 0.05 to 1 mM detection range using Si/SiO_2_/Au/uric acid-uric acid/prism. The device has a high sensitivity of 21.6/mM and a low detection limit of 0.02 mM.

Dopamine is a biological fluid that diffuses extensively throughout the human brain and is classified as a catecholamine [100, 101]. Dopamine is critical for the regulation of human movement and emotional responses [102]. As a result, the analysis of dopamine levels in the human body, remarkably the cerebrospinal fluid (CSF), is critical for the early detection and treatment of a variety of neural diseases [103, 104]. Pagano et al. recently proposed a novel approach for developing a dopamine detection system based on SERS-LSPR by fabricating a hybrid layer of perylene bisimide (PBI) derivative and multishaped Au nanostructures (AuNS) [45]. The fabricated detection system is capable of detecting dopamine concentrations as low as 1 × 10^−9^ mM over a detection range of 10^−9^—10^−5^ mM with a linear response of 10^−9^–10^−6^ M. Due to the coupling mechanism between localized (AuNS) and propagating surface plasmons in the PBI/AuNS hybrid layer, such low levels of dopamine can be detected. Additionally, PBI aids in the sensing device's surface functionalization. Eddin et al. modified the sensing surface of the Kretschmann-based SPR sensor by coating the Au thin film with chitosan-graphene quantum dots (CS-GQDs) to enable the detection of low levels of dopamine [27]. The sensing device detected dopamine with a sensitivity and limit of detection (LOD) of 0.011°/fM and 1 × 10^−12^ mM, respectively, over a detection range of 10^−12^—10^−9^ mM. It was discovered that the presence of CS-GQDs enhances the surface sensing ability to capture dopamine targets.

## 4. Summary and Further Perspectives

Kidneys are vital organs in the human body that filter the blood of metabolic waste and excess water while reabsorbing necessary chemicals and electrolytes, balancing acid-base conditions, and performing independent nephron functions. Thus, maintaining healthy kidneys is essential for human survival. However, kidney problems can result from physical damage or disease, resulting in various negative consequences and even death in humans. The concentration of urea and creatinine in either blood or urine could be used to diagnose kidney disease. The rise in normal urea and creatinine concentrations is a cause for concern that must be addressed. As a result, the development of SPR-based sensors is critical indetecting and monitoring kidney problems in the human body. Several researchers have published their research on this topic to develop advanced technology for several detection targets related to kidney problems early detection or monitoring.

For urea and creatinine detection, SPR-based sensors have been developed into three configurations: thin-film SPR, LSPR-based sensors, and optical fiber-based SPR sensors. The thin-film SPR generates the SPR phenomenon using either Kretschmann or other configuration. Typically, a sensing surface modification with ligands or an immobilization matrix is used to improve the sensing surfaces' analytes capturing mechanism, resulting in improved sensor performance. LSPR-based sensors typically use metal nanoparticles (MNPs) such as AuNPs or AgNPs at the SERS sensor surfaces to improve sensing ability by utilizing the plasmonic phenomenon. The presence of MNPs has been reported to improve sensitivity and LOD values. Finally, optical-based SPR sensors are fabricated by coating the outer layer of an optical fiber with a metal thin film to generate plasmonic waves from the SPR phenomenon. MNPs are also coated on the metal thin-film layer to improve sensor performance by combining the LSPR phenomenon coupled with the SPR phenomenon from the thin metal film.

The development of SPR-based sensors for the early detection of kidney disease is possible because SPR can be engineered to have a high sensitivity and a low LOD. As a result, SPRcan detect extremely low concentrations of targets such as urea, creatinine, glucose, uric acid, and dopamine in human secretion samples such as urine, saliva, and sweat. The use of human salivary secretion as a target detection method for kidney problems simplifies and accelerates the process. Salivary secretions are preferable to blood samples because they are easier to collect, have a lower demand, are easier to store, and are safer to handle. Saliva-based monitoring can also be used for bipolar disorders and continuous lactation. Therefore, a non-invasive sensor based on saliva biofluid has the potential to be developed for a variety of medical treatments.

## Figures and Tables

**Table 1:** Recent progress on SPR-based sensors for a variety of detection targets associated with kidney problems.

References	Sensor configuration	Analyte	Detection range	Sensitivity	Limit of detection (LOD)
[55]	Kretschmann: Au/nonenzymatic	Urea	50–800 mM	1.4 × 10^−3^ °/mM	—
	Kretschmann: Au/enzymatic (urease)	Urea	50–800 mM	1.62 × 10^−2^ °/mM	—
	Kretschmann: Au/nonenzymatic	Creatinine	10–200 mM	4 × 10^−3^ °/mM	—
	Kretschmann: Au/enzymatic (creatininase)	Creatinine	10–200 mM	1 × 10^−2^ °/mM	—
[57]	Kretschmann: Au	Creatinine	0.05–1 mM	11.6 cm^−2^ *μ*M	3.118 × 10^−2^ mM
	Kretschmann: Au/AgNPs (0 min after the mixture)	Creatinine	0.01–1 mM	21.2 cm^−2^ *μ*M	2.53 × 10^−3^ mM
	Kretschmann: Au/AgNPs (20 min after the mixture	Creatinine	0.01–1 mM	86.3 cm^−2^ *μ*M	1.9 × 10^−4^ mM
[28]	Kretschmann: Au/N-methacryloyl-(l)-histidine methyl ester (MAH)	Creatinine	1–100 mM	—	5.7 × 10^−2^—1.9 × 10^−1^ mM
[88]	LSPR : SERS with AuNPs inserted into the channel of BRDVD substrate	Urea	—	—	9.9 × 10^−3^ mM
		Creatinine	—	—	1.7 × 10^−3^ mM
[89]	LSPR : Au@MIL-101(Fe)	Creatinine	10^−3^—10^−1^ mM	—	1 × 10^−4^ mM
[56]	Optical fiber/Ag/Si—enzymatic	Urea	0–180 mM	—	—
[54]	Optical fiber/Au thin film/AuNPs— enzymatic	Urea	50 to 800 mM	0.06013 nm/mM	—
	Optical fiber/Au thin film/AuNPs—nonenzymatic	Urea	50 to 800 mM	0.04537 nm/mM	—
[42]	Optical fiber/Au thin film/GOD (covalent binding)	Glucose	0–2.8 mM	15.38 nm/mM	2.2 × 10^−3^ mM
[90]	Optical fiber/Au thin film/GOD (gel embedding)	Glucose	0–4.5 mM	2.52 nm/mM	1.2 × 10^−1^ mM
[72]	Kretschmann: Au–Cr (670 nm)	Glucose	4–12 mM	3.41 × 10^−3^ °/mM	4 mM
	Kretschmann: Au–Cr (785 nm)	Glucose	55–277 mM	2.73 × 10^−3^ °/mM	55 mM
[91]	Optical fiber/Ag/Si	Uric acid	0–0.9 mM	10.5 nm/mM	3.2 × 10^−3^ mM
[92]	LSPR—otto: Si/SiO2/Au/uricase-uric acid/prism	Uric acid	0.05–1 mM	21.6°/mM	2 × 10^−2^ mM
[44]	Kretschmann: Uric acid imprinted Poly(HEMA-MAC)- Fe^3+^NPs/Au/prism	Uric acid	2.9 × 10^−4^—2.3 × 10^−1^ mM	—	1.5 × 10^−4^ mM
[14]	LSPR : SERS with PIB/AuNS	Dopamine	10^−9^—10^−5^ mM	—	1 × 10^−9^ mM
[27]	Kretschmann: CS-GQDs/Au	Dopamine	10^−12^—10^−9^ mM	0.011°/fM	1 × 10^−12^ mM
[93]	Calorimetric LSPR sensors based on CDs-Au NPs	Dopamine	8.1 × 10^−4^—1.68 × 10^−2^ mM	—	2.3 × 10^−4^ mM
[94]	Optical fiber/AgNPs	Dopamine	2 × 10^−4^—3 × 10^−2^ mM	3 nm/*μ*M	2 × 10^−4^ mM
		Pneumophila	—	—	1

## Data Availability

No data were used in this study.
